# A cross-sectional study of opioid involvement in non-poisoning suicide – risks and prevention opportunities

**DOI:** 10.1186/s12889-021-10792-y

**Published:** 2021-04-21

**Authors:** Maryann Mason, Sarah B. Welch, Suzanne McLone, Tami Bartell, Patrick M. Lank, Karen Sheehan, Lori Ann Post

**Affiliations:** 1 Buehler Center for Health Policy and Economics, Feinberg School of Medicine, Northwestern University, 420 E. Superior St., Chicago, IL 60611 USA; 2 Smith Child Health Research, Outreach and Advocacy Center, Stanley Manne Children’s Research Institute, Ann & Robert H. Lurie Children’s Hospital of Chicago, 225 E. Chicago Ave., Chicago, IL 60611 USA; 3 Feinberg School of Medicine, Northwestern University, 420 E. Superior St., Chicago, IL 60611 USA

**Keywords:** Suicide, Opioids, Chronic health conditions, Substance misuse

## Abstract

**Objective:**

To examine prevalence, demographic, and incident factors associated with opioid-positivity in Illinois suicide decedents who died by causes other than poisoning.

**Method:**

Cross-sectional study of Illinois’ suicide decedents occurring between January 2015 and December 2017. Data come from the National Violent Death Reporting System. We used Chi-square tests to compare decedent and incident circumstance characteristics by opioid toxicology screen status. Incident narratives were analyzed to obtain physical and mental health histories and circumstances related to fatal injury events.

**Results:**

Of 1007 non-poisoning suicide decedents screened for opioids, 16.4% were opioid-positive. White race, age 75 and over, and widowed or unknown marital status were associated with opioid-positivity. Among opioid-positive decedents, 25% had a history of substance use disorder (SUD), 61% depression, and 19% anxiety. The majority (52%) of opioid-positive decedents died by firearm, a higher percentage than opioid-negative decedents.

**Conclusion:**

The opioid overdose crisis largely has not overlapped with non-poisoning suicide in this study. Overall, our analyses have not identified additional risk factors for suicide among opioid-positive suicide decedents. However, the overlap between opioid-positivity, SUD, and physical and mental health problems found among decedents in our data suggest several suicide prevention opportunities. These include medication assisted treatment for SUD which has been shown to reduce suicide, screening for opioid/benzodiazepine overlap, and limiting access to lethal means during opioid use. Improved death scene investigations for substances and use of the Prescription Drug Monitoring Program to document prescriptions are needed to further understanding of the role of substances in non-poisoning suicide.

## Background

The opioid misuse crisis has drawn attention to drug overdose deaths including unintentional and suicide poisoning deaths. In 2018, in the U.S., there were 46,802 opioid-involved overdose deaths [1]. Concurrent with the opioid crisis, the number and rate of deaths by suicide in the U.S. increased from 29,199 and 10.48 per 100,000 persons in 1999 to 48,344 and 14.21 per 100,000 in 2018 with wide variations among race, age and sex groups [2, 3]. At the same time, the proportion of suicide deaths across cause of death has shifted. From 1999 to 2018, the proportion of suicides by firearm decreased from 56.8 to 50.5%, the proportion of suicide deaths by suffocation increased from 18.5 to 28.6%, and the proportion of suicide deaths by poisoning decreased from 16.71 to 12.9% [3]. On the face of it, it would appear that the opioid misuse crisis has not intersected with suicide as signaled by the decline in suicide deaths due to poisoning. However, as it is well known that opioid use and misuse is associated with suicidal ideation and attempts [4], it is plausible that opioids may play a role in suicide deaths by causes other than poisoning.

This study goes beyond existing research on suicide and opioids to examine opioid involvement, as measured by the presence of opioids below the lethality threshold, in suicide decedents by causes other than poisoning, such as firearm and suffocation, which are leading causes of death among suicide decedents.

## Methods

This cross-sectional study uses Illinois data from the Center for Disease Control and Prevention’s National Violent Death Reporting System including suicide decedents with a date of death between January 1, 2015 and December 31, 2017 [5]. Data for the analysis includes all suicide decedents for which post mortem toxicology for the presence of opioids was conducted. Data were analyzed using SPSS Statistics (v. 26.0, Armonk, NY) descriptives and frequencies procedures to explore range and measures of central tendency. Chi-square statistics tested for associations between opioid positivity and demographic, non-opioid toxicology, and circumstance variables. Non-opioid toxicology variables included all non-opioid substances tested for in 70% or more of decedents. For variables with significant chi-square results, we ran comparisons of column proportion procedures to describe patterns in the data. We also reviewed narrative accounts of incidents for physical and mental health history and status at time of death and documentation of substances on scene to understand better their involvement in among opioid positive suicide decedents with causes of death other than poisoning.

## Results

The original data set included 2784 suicide decedents in 16 Illinois counties representing 65% of all Illinois suicide decedents from January 2015 to December 2017. We removed 1449 cases for which there were no toxicology testing or for which opioid testing was not included in post mortem toxicology, leaving 1335 cases. An examination of tested vs. not tested/testing results missing cases shows no statistically significant differences in the sex, age or race/ethnicity of those for whom toxicology reporting is available vs. those who were not tested or for whom testing data are missing. We also examined variation in opioid testing status and cause of death and found no significant differences by status (*p* = .779). Reasons we are aware of for not performing toxicology testing or not including opioid testing in toxicology vary by coroner/medical examiner office policies and practice, and include financial limitations, and infeasibility.

While we are primarily interested in opioid-involved non-poisoning suicide deaths, we explored demographic differences between poisoning and non-poisoning decedents. We found statistically significant differences in age and sex, with a larger proportion of poisoning deaths among those aged 45–54 (*p* = 0.05). A larger proportion of opioid positive females died by poisoning compared to other causes (*p* = 0.05).

**Fig. 1:**
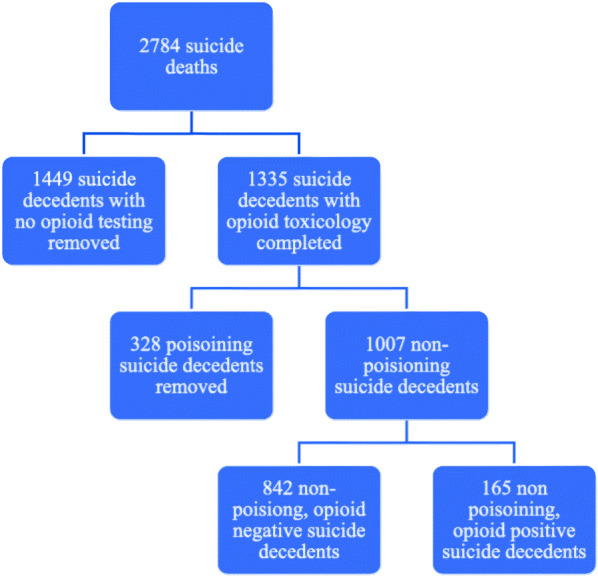
Study Inclusion Criteria

Because we are primarily interested in opioid involvement in non-poisoning suicide decedents, we removed 328 suicide cases where cause of death was poisoning (e.g., overdose) leaving 1007 cases for analysis. Of these 1007 suicide decedents, 83.6% (842) were negative and 16.4% (165) were positive for opioids. Figure 1 depicts study inclusion criteria. Opioid positive decedents were more likely to die by firearm and less likely to die by hanging, strangulation or suffocation than opioid negative decedents. See Table 1.

**Table 1:** Primary weapon by opioid toxicology

	Opioid positive % (n)	Opioid negative % (n)
Firearm	52.7 (87)*	39.7 (334)
Sharp instrument	6.7 (11)	4.0 (34)
Hanging, strangulation, suffocation	33.3 (55)	42.1 (354)*
Fall	*n* < 10	3.7 (31)
Explosive	*n* < 10	*n* < 10
Drowning	*n* < 10	2.5 (21)
Fire or burns	*n* < 10	*n* < 10
Motor vehicle including buses, motorcycles	*n* < 10	1.3 (11)
Other transport vehicle (e.g., trains, planes, boats	*n* < 10	5.6 (47)
Other (e.g., Taser, electrocution, nail gun)	*n* < 10	*n* < 10
Total	165	841

* *p* < 0.05

Compared to opioid negative suicide decedents, opioid positive suicide decedents have greater proportions of persons who were non-Hispanic White, 75 years old and older, and persons who were widowed or had unknown marital status at the time of their death. Opioid negative suicide decedents had greater proportions of decedents who were non-Hispanic Black, 15 to 24 years old, and had never married. See Table 2.

**Table 2:** Decedent demographics by opioid toxicology

	Opioid positive % (n)	Opioid negative % (n)
**Sex**		
Male	77.6 (128)	81.2 (683)
Female	22.4 (37)	18.8 (158)
**Race/Ethnicity**		
Non-Hispanic, White	90.3 (149)*	77.0 (648)
Non-Hispanic, Black	*n* < 10	10.5 (88)*
Am. Indian/Native Alaskan	0.0	*n* < 10
Asian/Pacific Islander	*n* < 10	3.0 (25)
Other (non-Hispanic)	0.0	*n* < 10
Two or more races	0.0	0.5 (4)
Hispanic	*n* < 10	8.8 (74)
**Age group**		
0–14	0.0	1.2 (10)
15–24	*n* < 10	17.3 (146)*
25–34	17.6 (29)	19.0 (160)
35–44	18.8 (31)	16.9 (142)
45–54	17.6 (29)	18.5 (156)
55–64	21.8 (36)	15.7 (132)
65–74	9.1 (15)	5.7 (48)
75+	11.5 (19)*	5.7 (48)
**Marital status**		
Married	32.7 (54)	28.1 (237)
Divorced	24.8 (41)	18.8 (158)
Separated	1.8 (3)	3.1 (26)
Never married	27.9 (46)	44.3 (373)*
Widowed	7.9 (13)*	3.8 (32)
Unknown	4.8 (8)*	1.9 (16)

* *p* ≤ 0.016

Testing positive for an opioid is associated with testing positive for one or more additional substances. Except for alcohol, a larger proportion of opioid positive decedents tested positive for each of the substances included in the analysis compared to opioid negative decedents. The mean number of substances present for those who were opioid positive was 2.5 vs. 0.68 for those who were opioid negative (*p* = 0.00). See Table 3.

**Table 3:** Toxicology by opioid presence

Substance present	Opioid positive % (n)	Opioid negative % (n)
Alcohol	32.0 (48)	34.3 (288)
Amphetamine	12.5 (17)*	5.1 (40)
Barbiturates	5.0 (7)*	0.1 (1)
Benzodiazepines	44.6 (68)*	12.0 (95)
Cocaine	9.5 (13)*	6.2 (15)
Marijuana	22.5 (34)*	13.2 (103)

*P ≤ 0.000

To learn more about substance use at time of death, we reviewed narrative data for the 165 opioid positive non-poisoning suicide decedents. Only 16.9% of opioid positive decedents had evidence of prescription medications noted in the case narrative. Few decedents had indications of consumption of large quantities of opioids proximate to their fatal injury. We also found few decedents in which evidence of drug use was found at the scene. See Table 4.

**Table 4:** Evidence of prescriptions/drugs at scene

	%(n)
Current prescription medications confirmed in incident narrative	16.9 (28)
No mention of prescription medications or drugs present on scene	72.7 (120)
Evidence of large quantity of drugs consumed (out of count prescriptions, etc.) in close proximity to death	< 10
Evidence of drugs at scene (illicit or prescription not confirmed in medical history)	< 10

A larger proportion of opioid positive decedents had a physical health problem that contributed to their death, a physical health crisis, and/or were experiencing a non-alcohol substance use disorder (SUD) at the time of their death compared to opioid negative suicide decedents. See Table 5.

**Table 5:** Selected circumstances by opioid positivity

	Opioid positive % (n)	Opioid negative % (n)
Physical health problem contributed to death	27.9 (46)*	10.3 (87)
Physical health problem was a crisis	9.7 (16)*	1.8 (15)
Had a non-alcohol related substance abuse problem^	24.8 (41)*	12.6 (106)
Prior suicide attempts	20.0 (33)	18.2 (143)
Left a suicide note	30.3 (50)	27.9 (235)
Disclosed suicidal thoughts/plans	17.0 (28)	15.4 (130)
Identified as having a current mental health problem	41.8 (69)	36.5 (316)

*P ≤ 0.000 ^now referred to as substance use disorder

To learn more about the medical issues faced by decedents we reviewed narrative data for the 165 opioid positive non-poisoning suicide cases. We found opioid positive decedents suffered from a variety of serious or chronic health problems including cancer (18%/29), heart disease (10%/17), hypertension (21%/34), and joint/back pain (15%/25). A substantial subset (23%/38) of these decedents had three or more serious health problems. The most frequent mental health issues were anxiety (19%/32) and depression (61%/101). About 17% of decedents had documented prescription medications at the time of fatal injury. A sub-set (9.2%) of narratives included mention of a recent hospitalization.

## Discussion

Opioid use is widespread in the U.S. In 2018, 18% of U.S. adults received an opioid prescription within the last year; 91% reported filling their prescription at least once [6]. In 2018, there were 45.2 opioid prescriptions dispensed per 100 people in Illinois [7]. A total of 3.6% of the U.S. population aged 12 and older reported misusing prescription pain relievers including opioids; 0.3% percent report using heroin in the past year [8]. Given widespread use and the well-known connection between opioid use and misuse and suicide ideation and attempts, we wondered about the extent of opioid involvement in suicide deaths by means other than overdose. To explore this, we used NVDRS data to examine opioid positivity in Illinois’ non-poisoning suicide deaths.

One-sixth (16.4%) of non-poisoning suicide decedents in our data set were positive for opioids. This tracks with national prescribing levels and reports of prescription pain reliever misuse and heroin use, but is low given Illinois’ opioid prescription rates. However, 52% of non-poisoning suicide decedents in our data set were not opioid tested. This low testing rate reflects coroner/medical examiner toxicology testing practices which vary by jurisdiction. Biases in perceptions of who uses opioids and financial constraints may influence testing practices. We hypothesize that the prevalence of opioids in our data set is an undercount due to testing practices. We expect that expanded testing would result in higher percentages of opioid-positive non-poisoning suicides.

The relatively low percentage of opioid-positive non-poisoning suicide deaths indicates that non-lethal opioid use is not as pervasive among suicide decedents who are tested post mortem for opioids as we anticipated. This is surprising given the extent of the opioid crisis in Illinois.

Opioid positivity among decedents in our data set is associated with White race, age 75 years and older, and firearm cause of fatal injury. We found higher prevalence of opioid-positive decedents with SUD and physical health problems compared to opioid-negative decedents. Opioid-positive decedents tested positive for more substances, including benzodiazepines, than did opioid-negative decedents. These findings are not surprising given known suicide risk factors. Overall, our analyses have not identified additional risk factors for suicide among opioid-positive suicide decedents. That said, our findings do point to several potential suicide prevention strategies for opioid users. One is the use of medications for opioid use disorder as medication can reduce suicide risk [9]. Benzodiazepine use is a known risk factor in self-harm and suicide [10, 11] and screening for opioid use in prescription of benzodiazepines and for benzodiazepine use when prescribing opioids may be helpful as a suicide prevention strategy even as it is not clear how this combination of substances contributes to suicidal behavior in non-poisoning suicide decedents.

Importantly, we lack a robust understanding of the role that opioids, and the mixture of substances decedents test positive for, play in suicides among this sub-set of decedents.

We found that opioids are not routinely used as a secondary means of lethality. Beyond this, it is unclear if opioid use among these suicide decedents is an artifact of medically prescribed usage, an indicator of an on-going SUD, or a means to reduce inhibitions prior to self-injury. We found relatively few cases in which evidence of drug use was present at the death scene suggesting opioid use was somewhat distal from the suicide injury event. Increased use of the Prescription Drug Monitoring Plan in death investigations could help determine if opioid positivity at time of death reflects medically prescribed therapeutic use or is reflective of misuse. Improved recording of prescriptions of all kinds at the death scene may improve our understanding of the context of opioid use in combination with other substances.

Opioid positive decedents are proportionally more likely to die by firearm than those who are opioid negative. The majority of opioid positive suicide decedents in our study died by firearm. This suggests that efforts to limit access to firearms, the most lethal of weapons, may be all the more important for those who are using opioids and with additional suicide risk factors such as physical health problems or substance misuse disorder.

This study uses Illinois data from the National Violent Death Reporting System (NVDRS). NVDRS is standardized and provides data for all 50 states, Puerto Rico and Washington DC. Our data are limited to Illinois and may not reflect the conditions present in other locations. Furthermore, while there is significant testing for opioids in this subsample, toxicology screening for opioids is not universal and 52% of cases were not included due to lack of toxicology testing. Lastly, due to the nature of suicide, third party decedent accounts may not accurately reflect the complete details of the incident.

## Conclusions

Our study findings suggest that opioid positivity is not common among non-poisoning suicide decedents despite the ubiquitous nature of the opioid crisis in the United States. Among non-poisoning suicide decedents, those who died by firearm had the highest prevalence of opioid positivity. This suggests a connection between firearm suicide and opioid use which should be further explored for prevention implications. Efforts to better understand the role opioids play in firearm suicide, e.g. if they are used to reduce inhibitions to self-harm, part of on-going SUD, or resulting from medical prescribed usage, can inform suicide prevention approaches.

## Data Availability

The dataset supporting the conclusions of this article is available in the WISQARS Fatal Injury and Violence repository, https://www.cdc.gov/injury/wisqars/fatal.html.
